# The Heterogeneous Metabolic Patterns of Ganglia in ^68^Ga-PSMA, ^11^C-choline, and ^18^F-FDG PET/CT in Prostate Cancer Patients

**DOI:** 10.3389/fonc.2021.666308

**Published:** 2021-04-23

**Authors:** Yiping Shi, Jian Guo Wu, Lian Xu, Yinjie Zhu, Yining Wang, Gan Huang, Jianjun Liu, Ruohua Chen

**Affiliations:** ^1^ Department of Nuclear Medicine, Ren Ji Hospital, School of Medicine, Shanghai Jiao Tong University, Shanghai, China; ^2^ Department of Nuclear Medicine, Second Affiliated Hospital, Nanchang University, Nanchang, China; ^3^ Department of Urology, Ren Ji Hospital, School of Medicine, Shanghai Jiao Tong University, Shanghai, China

**Keywords:** ^68^Ga-PSMA, ganglia, lymph node metastases, ^18^F-FDG, ^11^C-choline

## Abstract

**Purpose:**

Studies have indicated that PSMA-positive ganglia represent a diagnostic pitfall for nuclear medicine physicians. No studies have described choline and FDG uptake in ganglia, which may be a source of misdiagnosis. Herein, we described the percentage and uptake pattern of ^68^Ga-PSMA, ^11^C-choline and ^18^F-FDG PET/CT in ganglia and evaluated the heterogeneous metabolic patterns of ganglia to differentiate from lymph node metastases (LNM).

**Methods:**

Thirty-nine patients who underwent ^11^C-choline PET/CT and 120 patients who underwent ^68^Ga-PSMA PET/CT and ^18^F-FDG PET/CT were retrospectively analyzed. The prevalence of PSMA-positive, choline-positive and FDG-positive ganglia was determined, the SUVmax of ganglia in different locations were measured, and the configuration was described. The SUVmax cutoff of PSMA-PET, choline-PET and FDG-PET was determined by ROC curve analysis to differentiate ganglia from LNM.

**Results:**

329 PSMA-positive ganglia were identified in 120 patients, 95 choline-positive ganglia were identified in 39 patients, and 39 FDG-positive ganglia were identified in 34 patients. PSMA-positive uptake was observed in 98.3%, 95.8%, and 80.0% of cervical, coeliac, and sacral ganglia, respectively. Choline-positive uptake was observed in 84.6%, 97.4%, and 61.5% of cervical, coeliac, and sacral ganglia, respectively. FDG-positive uptake was observed in 16.7%, 13.3%, and 2.5% of cervical, coeliac, and sacral ganglia, respectively. Cervical and coeliac ganglia had a higher rate of PSMA-positive uptake than sacral ganglia. Choline uptake was highest in coeliac ganglia followed by cervical and sacral ganglia. PSMA, choline or FDG uptake in LNM was all significantly higher than ganglia. ROC curve analysis revealed that at a 4.1 SUVmax cutoff of PSMA-PET, the sensitivity, specificity and accuracy of LNM identification was 88.4%, 97.9% and 96.2%, respectively. ROC curve analysis revealed that at a 2.35 SUVmax cutoff for choline-PET, the sensitivity, specificity, and accuracy of LNM identification was 95.0%, 92.6% and 93.0%, respectively. ROC curve analysis revealed that at a 2.55 SUVmax cutoff for FDG-PET, the sensitivity, specificity, and accuracy of LNM identification was 77.3%, 87.2%, and 81.9%, respectively. PSMA-, Choline- and FDG-positive ganglia are mainly band-shaped; most LNMs exhibited nodular and teardrop-shaped configuration.

**Conclusion:**

^68^Ga-PSMA and ^11^C-choline uptake in ganglia was common, and FDG-positive ganglia were observed at lower frequency. Using ^68^Ga-PSMA, ^11^C-choline and ^18^F-FDG uptake and anatomic location and configuration, the differentiation of ganglia from adjacent LNM is feasible.

## Introduction

Prostate cancer is a common malignant tumor in males (1). Despite initial treatment, biochemical recurrence (BCR) is a problem after radical prostatectomy (2). The ability to determine the location and degree of recurrence of prostate cancer is important for guiding rescue treatment. However, conventional imaging techniques, including MRI and CT (3), have limited sensitivity. Since 2012, the application of functional positron emission tomography (PET) imaging, such as PSMA or choline PET, has significantly improved prostate cancer detection rates in BCR patients (4–8). PSMA PET has shown advantages in re-staging in BCR patients (9), as well as for the primary staging in initial diagnosed prostate cancer (10). Recently many studies have indicated that PSMA-positive ganglia represent a potential diagnostic pitfall for nuclear medicine physicians (11–14). The morphology and the PSMA uptake of the lesions along with delayed ^68^Ga-PSMA PET may be used to differentiate ganglia and lymph node metastases (LNM) (11, 12).

Besides ^68^Ga-PSMA PET, choline PET has also been commonly used to detect biochemical recurrence and has changed the management of BCR patients (15–17). ^11^C-choline was approved by the U.S. Food and Drug Administration in 2012 under an investigational new-drug application. However, choline-positive ganglia could be an important pitfall in prostate cancers, similar to PSMA-positive ganglia, which has not been reported in previous studies. ^18^F-FDG is the most widely used tracer in a variety of malignant tumors. ^18^F-FDG PET has been used in partial prostate cancers with a high Gleason grade, and specifically in prostate cancer patients with negative ^68^Ga-PSMA PET/CT findings (18–20). However, there have been no studies describing the patterns of FDG uptake in ganglia, either in prostate cancers or other malignant tumors. Therefore, whether there is choline and/or FDG uptake in ganglia, which could be a source of misdiagnosis, remains unclear and needs further investigation. In the present study, we described the percentage and uptake pattern of PSMA, choline and FDG in ganglia and evaluated the heterogeneous metabolic patterns of ganglia in order to differentiate from LNM.

## Methods

### Participants

The ethics committee of Renji Hospital approved the present retrospective study, and informed consent was waived. The present study was performed in accordance with the ethical standards as laid down in the 1964 Declaration of Helsinki and its later amendments. A total of 39 patients with prostate cancer who underwent ^11^C-choline PET/CT between March 2018 and December 2019 and 120 patients with prostate cancer who underwent ^68^Ga-PSMA and ^18^F-FDG PET/CT between July 2018 and August 2019 were enrolled. The patients’ characteristics, including age, Gleason grade score, PSA level and treatment history, were available for review.

### ^11^C-choline PET/CT and ^18^F-FDG PET/CT

^68^Ga-PSMA, ^11^C-choline and ^18^F-FDG were synthesized by our Radiochemistry Laboratory of Renji Hospital. Patients fasted for four hours before injecting 6.0 MBq/kg of ^11^C-choline and fasted for six hours before injecting 3.7 MBq/kg of ^18^F-FDG. The fasting blood glucose was lower than 14.0 mmol/L. Patients were required to rest for 60 minutes before undergoing ^18^F-FDG PET/CT. The PSMA ligand was ^68^Ga-PSMA-11. The injected dose of ^68^Ga-PSMA was 1.85 MBq/kg. ^68^Ga-PSMA PET/CT was scanned 55 minutes after injecting ^68^Ga-PSMA. Patients that underwent ^11^C-choline PET/CT were scanned 20 minutes after ^11^C-choline injection. PET/CT was carried out by a combined scanner (Biograph mCT). CT images (120 kV automatic milliamp current; 3 mm section thickness) were scanned from the patient’s upper thigh to the skull. PET was performed immediately after CT, and the acquisition time of each bed was three minutes.

### Image Evaluation

Two nuclear medicine physicians with eight to ten years of experience in PET/CT interpretation evaluated the image data together and resolved any disagreements by consensus. Regions of interest (ROI) were placed over the selected ganglia or lymph nodes metastases. The maximum standardized uptake value (SUVmax) was calculated as follows: maximum pixel value in the decay-corrected ROI activity (MBq/kg)/(the injected ^68^Ga-PSMA, ^11^C-choline or ^18^F-FDG radioactivity (MBq)/body weight (kg)).

Ganglia and adjacent LNM were grouped according to anatomic location, including cervical, coeliac, or sacral plexus. The main criteria for ganglia were focal ^68^Ga-PSMA, ^11^C-choline or ^18^F-FDG that projected onto a structure with typical type and location for sympathetic ganglia as previously described (11). Lesions were counted that were visually considered to be suggestive for ganglia or LNM exhibiting increased ^68^Ga-PSMA, ^11^C-choline or ^18^F-FDG tracer uptake relative to local background. The selected criteria for ganglia to avoid the introduction of possible bias were as follows: 1) A single ganglia (if more than one PSMA-, choline- or FDG-positive ganglia existed) with the highest ^68^Ga-PSMA, ^11^C-choline or ^18^F-FDG uptake was selected in each of the anatomic locations (cervical, coeliac, or sacral); 2) If the anatomic location had no PSMA-, choline- or FDG-positive ganglia, it was defined as PSMA-, choline- or FDG-negative. In addition, the same selected criteria for definite LNM with increased ^68^Ga-PSMA, ^11^C-choline or ^18^F-FDG uptake relative to local background were selected.

### Statistical Analysis

Results are either demonstrated as mean ± SD or as frequencies (%). For comparison of continuous variables, the 2-tailed unpaired Student t test was used. The x2 test was applied to compare nominal variables. All statistical analyses were performed using SPSS 21.0 (IBM Corp., USA), with a two-sided *P*<0.05 considered statistically significant.

## Results

### Prevalence of PSMA-Positive, Choline-Positive and FDG-Positive Ganglia

A total of 329 PSMA-positive ganglia were identified in 120 patients, 95 choline-positive ganglia were identified in 39 patients, and 39 FDG-positive ganglia were identified in 34 patients.

**Figure 1:**
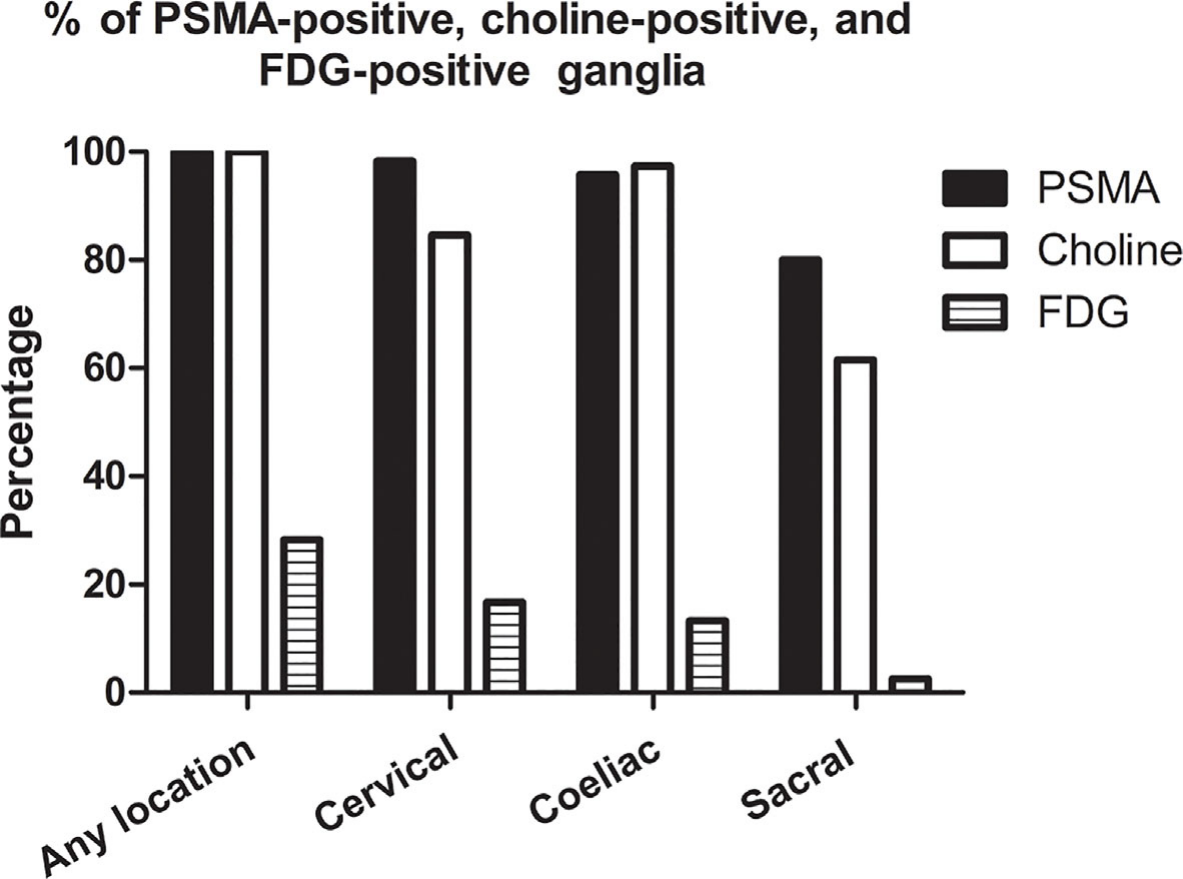
Frequencies of PSMA-positive, choline-positive and FDG-positive ganglia on per-patient-basis.

On a per-patient basis, 100% (120/120 patients) patients had positive PSMA uptake in ganglia (i.e., cervical, coeliac, or sacral). Grouped by location, PSMA-positive uptake was observed at a frequency of 98.3% (118/120 patients), 95.8% (115/120 patients), and 80.0% (96/120 patients) in cervical, coeliac, and sacral ganglia, respectively. Cervical and coeliac ganglia had a higher rate of PSMA-positive uptake than sacral ganglia (P<0.001 and P<0.001, respectively). Similar frequency of PSMA-positive uptake was observed between cervical and coeliac ganglia (P = 0.365) (**Figure 1**).

On a per-patient basis, 100% (39/39 patients) of patients had positive choline uptake in ganglia (i.e., cervical, coeliac, or sacral). Grouped by location, choline-positive uptake was observed at a frequency of 84.6% (33/39 patients), 97.4% (38/39 patients), and 61.5% (24/39 patients) in cervical, coeliac, and sacral ganglia, respectively (**Figure 1**). The frequency of choline-positive uptake was highest in coeliac ganglia followed by cervical and sacral ganglia (P < 0.05 for all pairs; **Figure 1**).

On a per-patient basis, 28.3% (34/120 patients) of patients had positive FDG uptake in ganglia (i.e., cervical, coeliac, or sacral). Grouped by location, FDG-positive uptake was observed at a frequency of 16.7% (20/120 patients), 13.3% (16/120 patients), and 2.5% (3/120 patients) in cervical, coeliac, and sacral ganglia, respectively. Cervical and coeliac ganglia had a higher rate of FDG-positive uptake than sacral ganglia (P<0.001 and P = 0.002, respectively). Similar frequency of FDG-positive uptake was observed between cervical and coeliac ganglia (P = 0.470) (**Figure 1**).

**Figure 2:**
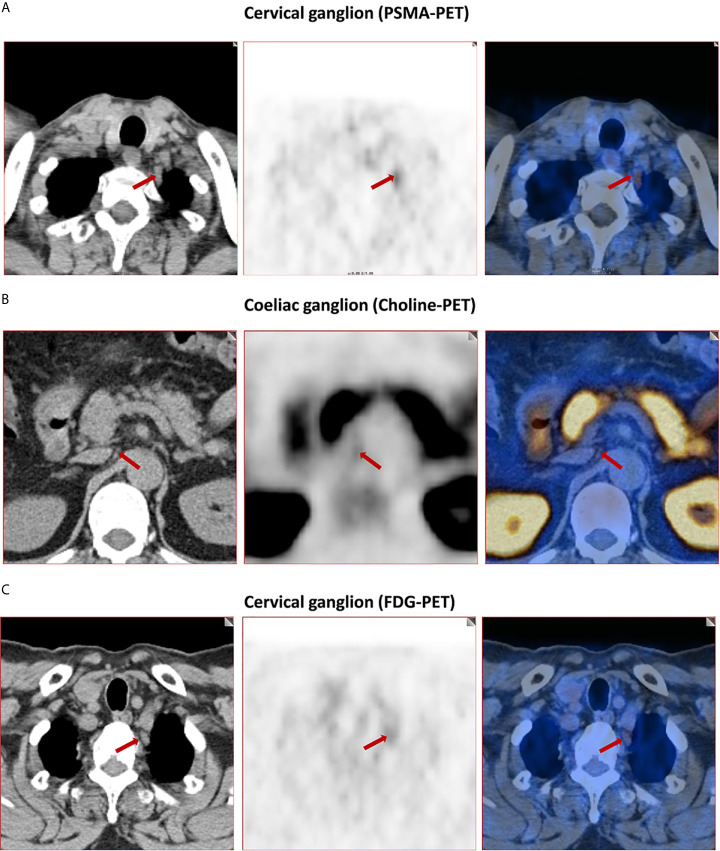
Representative images of PSMA-positive, choline-positive and FDG-positive ganglia. **(A)** PSMA-positive cervical ganglia (red arrow, 4.8 SUVmax for PSMA-PET). **(B)** Choline-positive coeliac ganglia (red arrow, 2.8 SUVmax for choline-PET). **(C)** FDG-positive cervical ganglia (red arrow, 2.9 SUVmax for FDG-PET).

The frequency of PSMA-positive and choline-positive ganglia per-patient were 100%, but both were significantly higher than the prevalence of FDG-positive (100% vs. 28.3%, and 100% vs. 28.3%, respectively, P < 0.001 for both) (**Figure 1**). Representative PSMA-positive, choline-positive and FDG-positive ganglia were shown in **Figure 2**.

### Absolute Uptake of Ganglia

**Figure 3:**
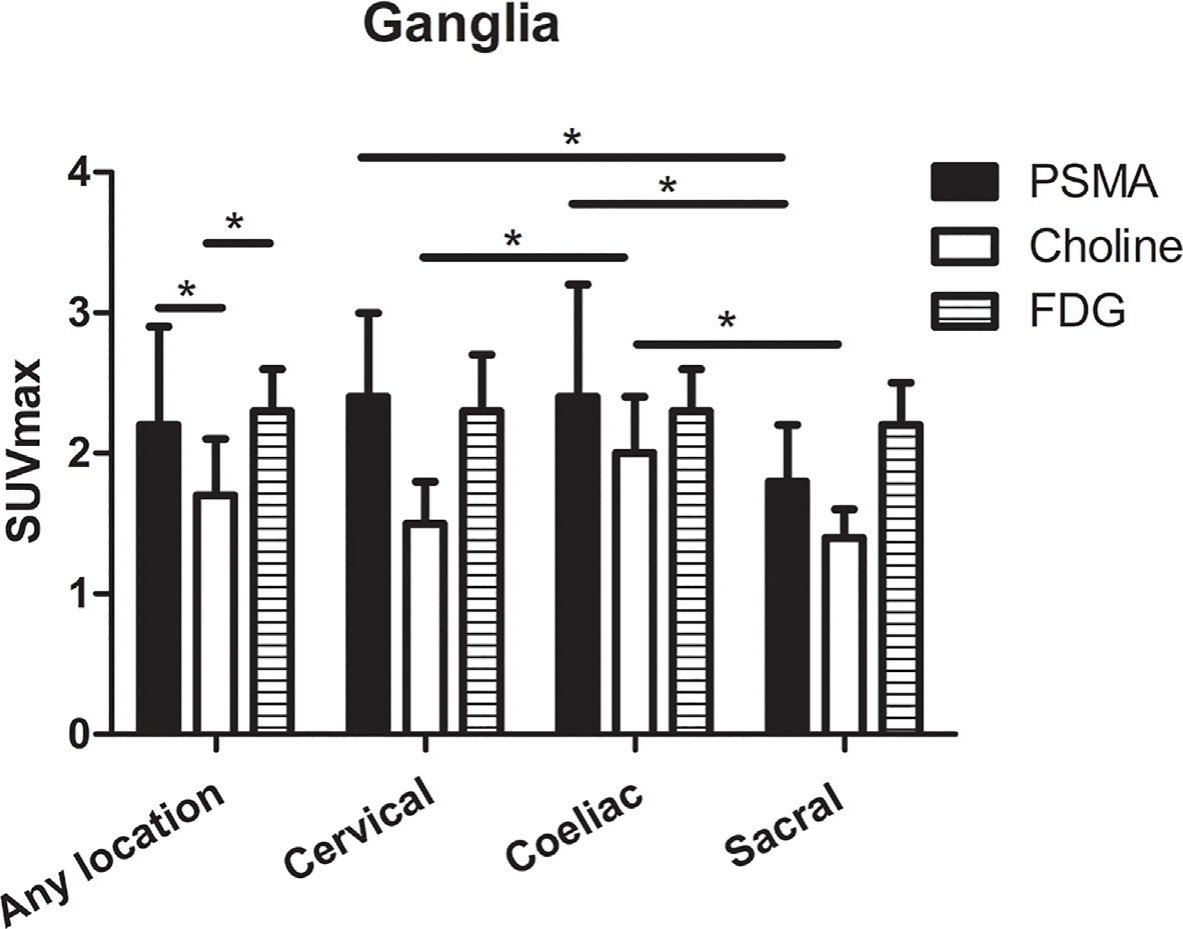
The SUVmax of PSMA-PET, choline-PET and FDG-PET at any location, and cervical, coeliac, and sacral ganglia. *P < 0.05.

For qualitative analysis, the SUVmax of PSMA-PET ranged from 1.3 to 6.6. No significant difference was observed in PSMA uptake between ganglia in cervical and coeliac (2.4 ± 0.6 *vs*. 2.4 ± 0.8, P=0.366). However, PSMA uptake in cervical (2.4 ± 0.6 *vs*. 1.8 ± 0.4, P<0.001) and coeliac ganglia (2.4 ± 0.8 *vs*. 1.8 ± 0.4, P<0.001) were both significantly higher than in sacral ganglia (**Figure 3**). For qualitative analysis, the SUVmax of choline-PET ranged from 1.1 to 2.8. choline uptake was highest in coeliac ganglia followed by cervical and sacral ganglia (coeliac was 2.0 ± 0.4; cervical was 1.5 ± 0.3; sacral was 1.4 ± 0.2; P < 0.05 for all pairs). The SUVmax of FDG-PET ranged from 2.0 to 3.5. No significant difference was observed in FDG uptake among cervical, coeliac and sacral ganglia(P =0.915, **Figure 3**).

The SUVmax of choline-PET was lower than the SUVmax of PSMA-PET (1.7 ± 0.4 *vs*. 2.2 ± 0.7, P < 0.001) and SUVmax of FDG-PET (1.7 ± 0.4 *vs*. 2.3 ± 0.3, P < 0.001), and no significant difference was observed between SUVmax of PSMA-PET and FDG-PET (P=0.668). The detailed SUVmax for ganglia are listed in **Table 1**.

**Table 1:** SUVmax of PSMA-PET, choline-PET and FDG-PET in ganglia.

Parameter		Ganglia	Cervical	Coeliac	Sacral
		Any Location			
**PSMA**	Mean	2.2	2.4	2.4	1.8
	SD	0.7	0.6	0.8	0.4
	Median	2.1	2.4	2.2	1.7
	Range	1.3-6.6	1.3-4.4	1.3-6.6	1.3-3.3
**Choline**	Mean	1.7	1.5	2	1.4
	SD	0.4	0.3	0.4	0.2
	Median	1.6	1.5	2	1.4
	Range	1.1-2.8	1.1-2.3	1.4-2.8	1.1-1.7
**FDG**	Mean	2.3	2.3	2.3	2.2
	SD	0.3	0.4	0.3	0.2
	Median	2.1	2.1	2.1	2.3
	Range	2.0-3.5	2.0-3.5	2.0-2.8	2.0-2.3

### Comparison of SUVmax of Choline-PET and FDG-PET Between Ganglia and LNM

**Figure 4:**
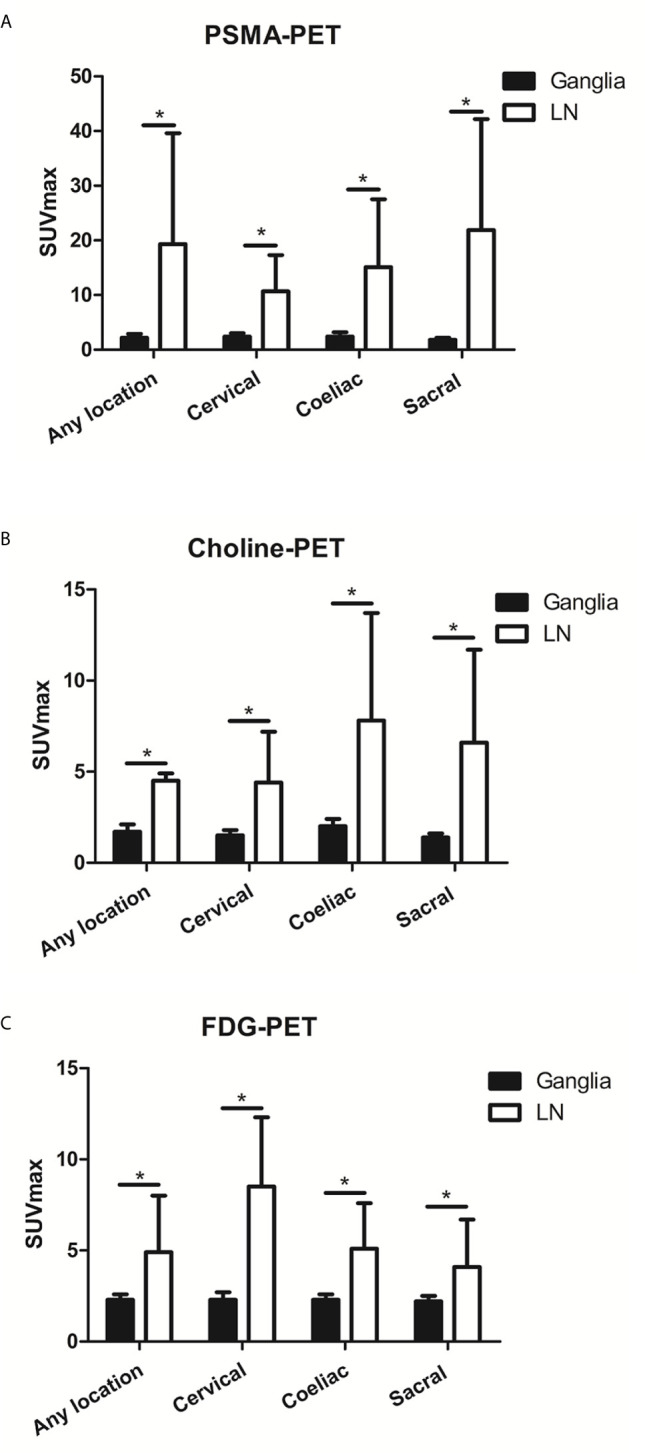
The PSMA, choline and FDG uptake in ganglia and adjacent LNM. **(A)** The PSMA uptake in ganglia and adjacent LNM. **(B)** The choline uptake in ganglia and adjacent LNM. **(C)** The FDG uptake in ganglia and adjacent LNM. *P < 0.05.

On a per-patient basis, PSMA-positive lymph node metastases in any location (i.e., cervical, coeliac, or sacral) were detected in 40.8% (49/120 patients). Grouped by anatomy, PSMA-positive lymph nodes metastases were found at a frequency of 6.7% (8/120 patients), 10.8% (13/120 patients), and 40.0% (48/120 patients) near the typical location of cervical, coeliac, and sacral ganglia, respectively. Frequencies between the occurrence of PSMA-positive ganglia and lymph node metastases were different for any and each separate location (P < 0.001). PSMA uptake in lymph node metastases was significantly higher than in ganglia for any location, cervical, coeliac, and sacral locations (any location:19.3 ± 19.0 *vs*. 2.2 ± 0.7, P < 0.001; cervical: 10.7 ± 6.6 *vs*. 2.4 ± 0.6 0.7, P < 0.001; coeliac: 15.1 ± 12.4 *vs*. 2.4 ± 0.8, P < 0.001; sacral: 21.9 ± 20.3 *vs*. 1.8 ± 0.4, P < 0.001) (**Figure 4A**).

Choline-positive LNM in any location was detected in 35.9% (14/39) of patients. Choline-positive LNM was found at a frequency of 5.1% (2/39) of patients at the cervical location, 12.8% (5/39) of patients at the coeliac location, and 33.3% (13/39) of patients at the sacral location. Frequencies in the occurrence of choline-positive ganglia and LNM were significantly different at each location (P < 0.001). Choline uptake in LNM was significantly higher than in ganglia for cervical, coeliac, and sacral locations (any location: 6.6 ± 5.1 *vs*. 1.7 ± 0.4, P < 0.001; cervical: 4.5 ± 0.4 *vs*. 1.5 ± 0.3, P < 0.001; coeliac: 4.4 ± 2.8 *vs*. 2.0 ± 0.4, P < 0.001; sacral: 7.8 ± 5.9 *vs*. 1.4 ± 0.2, P < 0.001) (**Figure 4B**).

FDG-positive LNM at any location (i.e., cervical, coeliac, or sacral) was detected in 25.8% (31/120) of patients. FDG-positive LNM was found at a frequency of 5.0% (6/120) of patients, 6.7% (8/120) of patients, and 25.0% (30/120) of patients near the typical location of cervical, coeliac, and sacral ganglia, respectively. Frequencies in the occurrence of FDG-positive ganglia and LNM were significantly different at each location (P < 0.001). FDG uptake in LNM was significantly higher than in ganglia cervical, coeliac, and sacral locations (any location: 4.9 ± 3.1 *vs*. 2.3 ± 0.3, P < 0.001; cervical: 8.5 ± 3.8 *vs*. 2.3 ± 0.4, P < 0.001; coeliac: 5.1 ± 2.5 *vs*. 2.3 ± 0.3, P < 0.001; sacral: 4.1 ± 2.6 *vs*. 2.2 ± 0.3, P < 0.001) (**Figure 4C**).

### ROC Curve Analysis to Differentiate Ganglia From LNM

**Figure 5:**
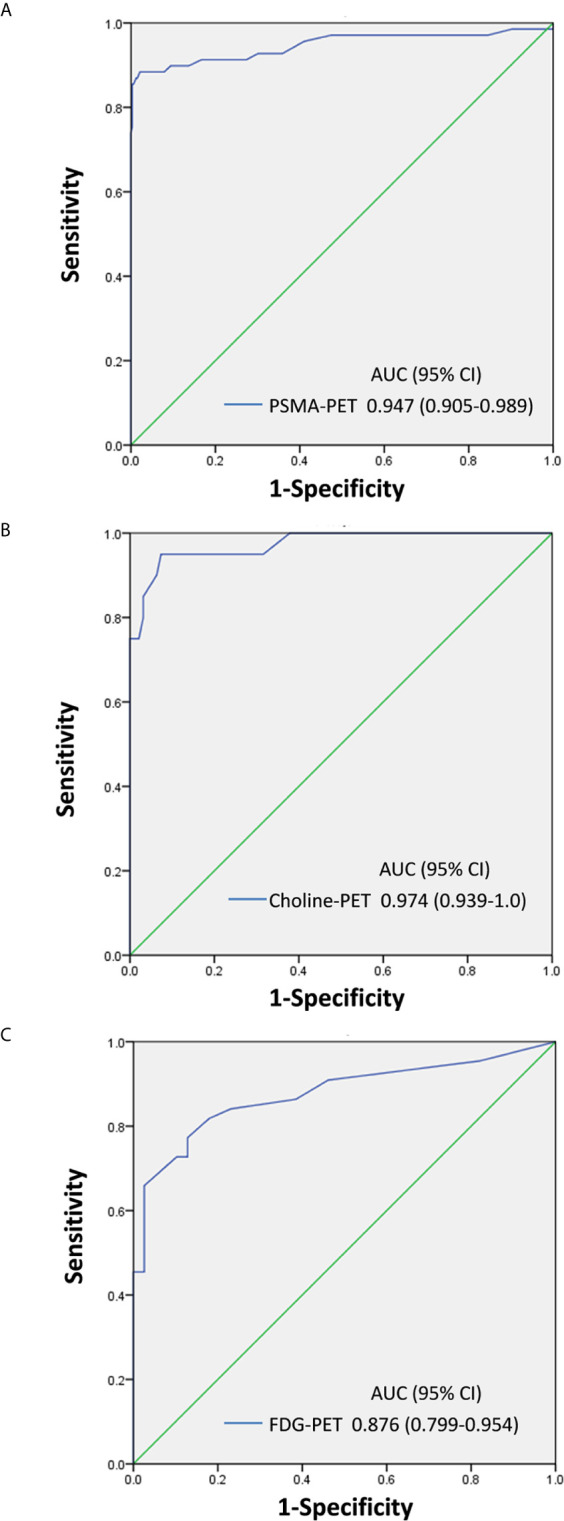
SUVmax of PSMA-PET, choline-PET and FDG-PET for distinguishing between LNM and ganglia. **(A)** PSMA-PET receiver-operating characteristic (ROC) curve analysis showing sensitivity, specificity, and area under curve. **(B)** Choline-PET receiver-operating characteristic (ROC) curve analysis showing sensitivity, specificity, and area under curve. **(C)** FDG-PET receiver-operating characteristic (ROC) curve analysis showing sensitivity, specificity, and area under curve.

The optimal SUVmax threshold of PSMA-PET, choline-PET or FDG-PET for distinguishing between LNM and ganglia is shown in **Figure 5**. Receiver-operating characteristic (ROC) curve analysis revealed that at when the SUVmax cutoff of PSMA-PET was 4.1, the sensitivity, specificity and accuracy for identifying lymph node metastasis were 88.4% (61/69), 97.9% (322/329) and 96.2% (383/398), respectively (**Table 2**). And the area under curve was 0.947(95%CI: 0.905-0.989). 2.1% (7/329) of ganglia had a SUVmax of PSMA-PET more than 4.1.

**Table 2:** Identification efficiency between lymph node metastasis and ganglia.

Parameter	Threshold	AUC (95% CI)	Sensitivity	Specificity	Accuracy
PSMA-PET	4.10	0.947(0.905-0.989)	88.4%	97.9%	85.9%
Choline-PET	2.35	0.974(0.939-1.0)	95.0%	92.6%	93.0%
FDG-PET	2.55	0.876 (0.799-0.954)	77.3%	87.2%	81.9%

Similarly, ROC curve analysis revealed that when the SUVmax cutoff of choline-PET was 2.35, the sensitivity, specificity, and accuracy for identifying LNM was 95.0% (19/20), 92.6% (88/95), and 93.0% (107/115), respectively. The area under the curve was 0.974 (95%CI: 0.939-1.0). In addition, 7.4% of ganglia showed a SUVmax of choline-PET higher than 2.35.

ROC curve analysis also revealed that at a 2.55 SUVmax cutoff for FDG-PET, the sensitivity, specificity, and accuracy for identifying LNM was 77.3% (34/44), 87.2% (34/39), and 81.9% (68/83), respectively. The area under the curve was 0.876 (95%CI: 0.799-0.954). In addition, 12.8% of ganglia showed a SUVmax of FDG-PET higher than 2.55.

### Comparison of Anatomic Morphology Between Ganglia and LNM

**Figure 6:**
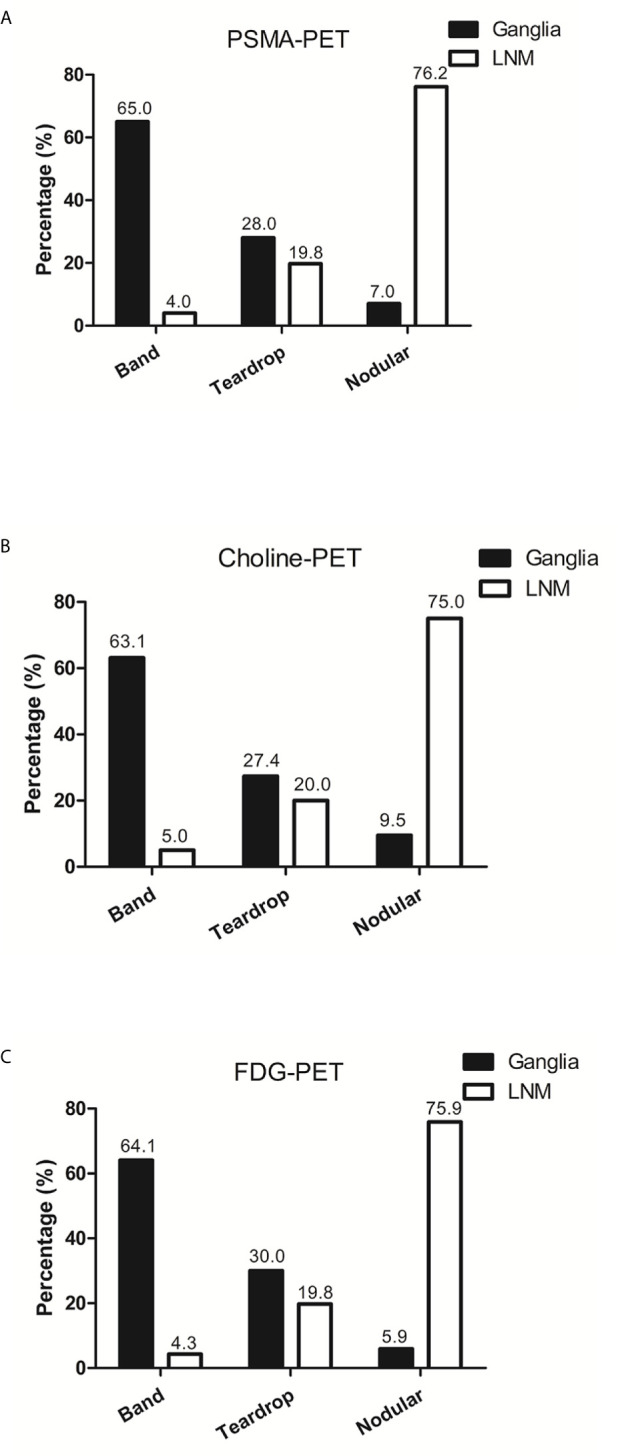
The anatomic morphology of ganglia and LNM in PSMA-PET, choline-PET and FDG-PET. **(A)** The anatomic morphology of ganglia and LNM in PSMA-PET. **(B)** The anatomic morphology of ganglia and LNM in choline-PET. **(C)** The anatomic morphology of ganglia and LNM in FDG-PET.

We further analyzed the anatomic morphology in ganglia and LNM. In PSMA-positive ganglia, 65.0% exhibited a band-shaped configuration, 28.0% exhibited a teardrop-shaped configuration, and 7.0% exhibited a nodular configuration. In PSMA-positive LNM, only 4.0% exhibited a band-shaped configuration, 19.8% exhibited a teardrop-shaped configuration, and 76.2% exhibited a nodular configuration (P < 0.001; **Figure 6A**).

In choline-positive ganglia, 63.1% exhibited a band-shaped configuration, 27.4% exhibited a teardrop-shaped configuration, and 9.5% exhibited a nodular configuration. In choline-positive LNM, only 5.0% exhibited a band-shaped configuration, 20.0% exhibited a teardrop-shaped configuration, and 75.0% exhibited a nodular configuration (P < 0.001; **Figure 6B**).

In FDG-positive ganglia, 64.1% exhibited a band-shaped configuration, 30.0% exhibited a teardrop-shaped configuration, and 5.9% exhibited a nodular configuration. In FDG-positive LNM, 4.3% exhibited a band-shaped configuration, 19.8% exhibited a teardrop-shaped configuration, and 75.9% exhibited a nodular configuration (P < 0.001; **Figure 6C**).

## Discussion

Many studies have indicated that PSMA-positive ganglia represent a potential diagnostic pitfall for nuclear medicine physicians. To solve this problem, some strategies have been proposed, including carefully anatomic correlation and compare and examine the morphology of the lesions (11). Recently, Ian et al. showed that delayed ^68^Ga-PSMA PET/CT can be used to differentiate between ganglia and LNM (12). The previous studies reporting PSMA uptake by ganglia may help us to differentiate LNM from ganglia. However, choline and ^18^F-FDG are two additional tracers that are widely used in prostate cancer patients. Nonetheless, there have been no studies describing the patterns of choline and FDG uptake in ganglia.

In the current study, we evaluated the metabolic pattern of ^68^Ga-PSMA, ^11^C-choline and ^18^F-FDG uptake in cervical, coeliac, and sacral ganglia using ^68^Ga-PSMA, ^11^C-choline and ^18^F-FDG PET/CT and compared the findings and parameters with LNM. Our study was the first to describe ^68^Ga-PSMA, ^11^C-choline and ^18^F-FDG uptake patterns at different locations (cervical, coeliac, and sacral). Our results showed that ganglia at different location have heterogeneous ^68^Ga-PSMA, ^11^C-choline uptake intensity and homogeneous ^18^F-FDG uptake intensity. In addition, we performed a systematic comparison of the different ganglia and adjacent LNM and demonstrated that ^68^Ga-PSMA, ^11^C-choline and ^18^F-FDG uptake is higher in LNM. In addition, LNM showed a clearly different configuration compared with ganglia. Furthermore, we used ROC curve analysis to differentiate LNM from ganglia and found preferable sensitivity, specificity, and accuracy in ^68^Ga-PSMA, ^11^C-choline and ^18^F-FDG PET/CT.

In this study, we identified PSMA-positive ganglia in 100% of our patients, 98.3% in cervical, 95.8% in coeliac and 80.0% in sacral ganglia, which were similar with the PSMA-positive rates found by Christoph (11). Though lymph node metastases had a statistically significant higher SUVmax of PSMA-PET than ganglia, there was a significant overlap in SUVmax of PSMA-PET between lymph node metastases and ganglia. Vinsensia et al. previously suggested SUVmax 2.0 of PSMA-PET as the threshold for PSMA-positive lymph nodes (21). However, our study demonstrated that 59.9% of ganglia had a SUVmax of PSMA-PET more than 2.0. In the current study, we sought to determine the optimal threshold of SUVmax of PSMA-PET for predicting lymph node metastases. ROC analysis demonstrated that the highest accuracy (96.2%) was obtained with the SUVmax of PSMA-PET 4.1, and the sensitivity and specificity for identifying lymph node metastasis were 88.4%, 97.9%, respectively. As we all known, this is the first study to determine the optimal threshold of SUVmax of PSMA-PET by ROC analysis for identifying lymph node metastases from ganglia, and our diagnostic efficiency is higher than that of other studies (12). When we use 4.1 as the cut-off, only 2.1% of ganglia had a SUVmax of PSMA-PET more than 4.1.

Besides PSMA-positive ganglia which have been described in previous studies, we also investigated in detail the choline and FDG uptake in ganglia which have never been reported before. We identified choline-positive ganglia in 100% of patients, including 84.6% in cervical, 97.4% in coeliac ganglia, and 61.5% in sacral ganglia. Though the frequency of choline-positive ganglia was high, the range of SUVmax of choline-PET in ganglia was narrow, which ranged from 1.1 to 2.8. However, the range of SUVmax of choline-PET in LNM was high, which ranged from 1.8 to 19.0. In addition, choline uptake in LNM was significantly higher than in ganglia for all locations, including cervical, coeliac, and sacral locations. ROC curve analysis revealed that when the SUVmax cutoff of choline-PET was 2.35, it showed preferable sensitivity, specificity, and accuracy for identifying LNM. Only 7.4% of ganglia had a SUVmax of choline-PET higher than 2.35. In addition, choline uptake was highest in coeliac ganglia followed by cervical and sacral ganglia. It should be noted that the SUVmax of sacral ganglia was low (1.7), thus when we differentiated LNM from ganglia in choline-PET, the lesion location was considered and helped us to better distinguish between them. Although the typical location of the ganglia, knowledge about anatomy and disease stage helps in differentiation between LNM and ganglia, the additional SUVmax cut-off value is rarely used. This is mainly due to PSMA is highly sensitive and can also detect LNM as small as 2-3 mm which may show only faint PSMA uptake (hence lower SUVmax). In this LNM with faint PSMA, it is not feasible to differentiate between LNM and ganglia according to the SUVmax.

^18^F-FDG PET is widely used in many malignant tumors. However, FDG-positive ganglia have not been described in previous studies. In the present study, we identified FDG-positive ganglia in 28.3% of patients, including 16.7% in cervical, 13.3% in coeliac ganglia, and 2.5% in sacral ganglia, which were lower than the frequency of choline-positive ganglia. In addition, choline uptake in LNM was significantly higher than in ganglia for all locations, including cervical, coeliac, and sacral locations. When we used 2.55 SUVmax as a cut-off, 12.8% of ganglia had a SUVmax using FDG-PET of more than 2.55. In addition, though no significant difference was observed in FDG uptake for ganglia in different locations, the frequency of FDG-positive sacral lesion in ganglia was 2.5% but was 25% in LNM. Furthermore, the range of SUVmax of FDG-PET in ganglia was narrow and ranged from 2.0 to 3.5; the maximum of sacral ganglia was 2.3. Lymph nodes are most likely to metastasize into the sacral location and identification is more often required compared with cervical and coeliac location. Therefore, the FDG-PET SUVmax range of sacral ganglia and the FDG-positive rates between ganglia and LNM should be determined, which helped to better distinguish them.

In addition to the ^68^Ga-PSMA, ^11^C-choline and ^18^F-FDG uptake difference, we evaluated the morphology differences between ganglia and LNM. PSMA-, Choline- and FDG-positive ganglia are more often band-shaped. However, most LNMs exhibited nodular configuration or teardrop-shaped configuration. In several lesions with teardrop-shaped structure, ^68^Ga-PSMA, ^11^C-choline and ^18^F-FDG uptake and anatomic location was used to differentiate ganglia from LNM. ^68^Ga-PSMA, ^11^C-choline and ^18^F-FDG uptake characteristics, anatomic location, and configuration can be used to differentiate between ganglia from adjacent LNM.

Although our results indicated different heterogeneous characteristics of tracer uptake in ^68^Ga-PSMA, ^11^C-choline or ^18^F-FDG, the mechanism remains unclear. Many studied reported that peripheral nerve ganglia uptake PSMA (11). It has been reported that astrocytes express PSMA physiologically as PSMA is related to their homologue glutamic acid carboxypeptidase III (22, 23). The uptake of ^18^F-FDG in ganglia was correlated with the expression level of glucose transporter-1 and glucose transporter-3 (24). Thus, the heterogeneous metabolic patterns of ganglia may be attributed to the heterogeneous expression of PSMA, glucose transporter-1 and glucose transporter-3 and choline content.

Our study had several limitations. The definition of LNM and ganglia has been mainly based on characteristic imaging features, such as typical anatomic location. However, pathological evidence is not feasible in the clinic because of ethical and practical reasons. The SUVmax cut-off of PSMA-PET, choline-PET and FDG-PET could differentiate LNM from ganglia. However, the SUVmax threshold may be influenced by different PET/CT scanner models, the PSMA or choline ligand, the scan procedure, etc. Therefore, in the clinical setting, it is essential to establish the optimal SUVmax cut-off according to the actual imaging conditions, instead of arbitrarily using the threshold of the current study. In addition, choline-PET and PSMA/FDG-PET were derived from different group of patients, so the comparison between them might be unfair due to the selection bias. Furthermore, the present study had a relatively small sample size and it is a retrospective study. Therefore, the results might have been influenced by selection bias and should be cautiously interpreted. Further prospective studies with more patients are required to confirm our study.

## Conclusions

The current study is the first to describe the patterns of ^11^C-choline and ^18^F-FDG uptake in ganglia. ^68^Ga-PSMA and ^11^C-choline uptake in ganglia was very common, and FDG-positive ganglia were observed at a lower frequency compared with PSMA-positive and choline-positive ganglia. ^68^Ga-PSMA, ^11^C-choline and ^18^F-FDG uptake characteristics, anatomic location, and configuration may be used to differentiate between ganglia from adjacent LNM.

## Data Availability Statement

The raw data supporting the conclusions of this article will be made available by the authors, without undue reservation.

## Ethics Statement

The studies involving human participants were reviewed and approved by RenJi Hospital. Written informed consent for participation was not required for this study in accordance with the national legislation and the institutional requirements.

## Author Contributions

RC and JL designed and wrote the experiments. YS, JW, LX, YW and GH collected and analyzed the data. YZ revised the manuscript. All authors contributed to the article and approved the submitted version.

## Funding

This work was supported by grants from the National Natural Science Foundation of China (nos. 81701724, 81771858, 81830052, 81771861), Shanghai Advanced Appropriate Technology Promotion Projects (no. 2019SY029) and JiangXi Provincial Department of Science and Technology (Grant Number: 20161BBG70202 & 20071BBG70050), Jiangxi Provincial Health Commission (Grant Number: 20161071 & 20171090).

## Conflict of Interest

The authors declare that the research was conducted in the absence of any commercial or financial relationships that could be construed as a potential conflict of interest.
